# A Fossilized Energy Distribution of Lightning

**DOI:** 10.1038/srep30586

**Published:** 2016-07-28

**Authors:** Matthew A. Pasek, Marc Hurst

**Affiliations:** 1 School of Geoscience, NES 204, University of South Florida, 4202 E Fowler Ave Tampa FL 33620, USA; 2 Independent Geological Services, Inc. 4432 Burlington Drive, Winter Haven, FL 33880, USA.

## Abstract

When lightning strikes soil, it may generate a cylindrical tube of glass known as a fulgurite. The morphology of a fulgurite is ultimately a consequence of the energy of the lightning strike that formed it, and hence fulgurites may be useful in elucidating the energy distribution frequency of cloud-to-ground lightning. Fulgurites from sand mines in Polk County, Florida, USA were collected and analyzed to determine morphologic properties. Here we show that the energy per unit length of lightning strikes within quartz sand has a geometric mean of ~1.0 MJ/m, and that the distribution is lognormal with respect to energy per length and frequency. Energy per length is determined from fulgurites as a function of diameter, and frequency is determined both by cumulative number and by cumulative length. This distribution parallels those determined for a number of lightning parameters measured in actual atmospheric discharge events, such as charge transferred, voltage, and action integral. This methodology suggests a potential useful pathway for elucidating lightning energy and damage potential of strikes.

Cloud-to-ground lightning is a high-energy phenomenon that transfers electric charge, either positive or negative, between the atmosphere and the surface. Voltages associated with lightning strikes may exceed 10^8^ V, and the total energy of a strike may reach 10^9^J, heating the air to temperatures exceeding 30,000 K^1^. This phenomenon is associated with several detrimental effects including human fatalities, forest fires, and property damage, and a few positive effects such as natural nitrogen fixation^2^ and production of reduced oxidation state phosphorus compounds that may improve the solubility of phosphorus in soil^3^.

When cloud-to-ground lightning strikes an appropriate target material such as sand, soil, rock, or clay, the current flows through the target, heating the material to temperatures that exceed its vaporization point, followed by rapid cooling that results in quenching to form a hollow cylinder of glass known as a fulgurite (Fig. 1). The morphology of fulgurites is strongly dependent on material composition^4^,^5^,^6^, and for natural fulgurites, the thickness of the glass is inversely related to fraction of material composed of SiO_2_. Lightning is a ubiquitous phenomenon on Earth, with a global flash rate of about 45 times per second^7^, a majority (75–90%) of which occur over continental landmass^8^. About a quarter of these strikes occur from a cloud to the ground^9^, and hence the number of potential fulgurite-forming events is significant, with up to 10 fulgurites formed globally per second. This estimate depends on the efficiency of fulgurite formation by lightning, which is highest when striking barren sand, soil, or rock^10^. Lightning striking forested areas may not necessarily form a fulgurite, though fulgurites are known to form when lightning strikes trees^11^. Fulgurites have also been known to form associated with human-made objects such as steel pylons, where the metallic structure may actually induce lightning to travel along its length^12^.

Fulgurites that are formed in a homogenous target may preserve the probability distribution of a key lightning parameter—the energy of a strike—in their morphology. To this end, we determined the relationship between numerical frequency and energy of cloud-to-ground lightning strikes by studying a set of 266 fulgurites from two quartz sand mines in Polk County, Florida, USA (Fig. 2). We then compared these results to predicted distributions calculated using simulated energy and length parameters, coupled to a simple fracture model. Our motivating hypothesis within this work is that fulgurites might also follow a lognormal distribution with respect to energy, as many other lightning parameters follow lognormal distributions.

## Results

The fulgurites were arranged according to decreasing diameter. Each fulgurite was given a number corresponding to the number of fulgurites equal to or larger than its diameter. Correspondingly, the frequency distribution of the fulgurites may be calculated by ordering the fulgurites in terms of energy per length (MJ/m) and then from 1) the length of each fragment with cumulative length becoming the dependent variable, or 2) by using total number as the dependent variable. Alternatively, the distribution of total energy (MJ/m × length) to total number can also be used to provide a distribution. These frequency diagrams are given as Fig. 3. All three methods generate linear relationships between the log of energy (MJ/m or total MJ) vs. frequency (number of occurrences, or cumulative length).

We also prepared simulations of fulgurite distributions that might arise from specific energy distributions. We find that lognormal distributions of energy per length with a normal distribution or constant length of each fulgurite are most consistent with the natural fulgurite distributions (see supporting information). These simulations help to provide some confidence in the approaches taken below.

Three approaches (energy per meter vs. cumulative length, energy per meter vs. cumulative number, total energy vs. cumulative number) were chosen to allow for multiple approaches to the estimating lightning energy distributions. Cumulative length is used as it allows for a direct comparison to frequency of events for a fractured material in which all parts may not be recovered. There is little change in fulgurite diameter across the length of a fulgurite^1^, even if the fulgurite has a total length greater than one meter. Hence, two fragments of the same fulgurite should have the same diameter. To this end, comparing energy to cumulative length helps eliminate the possibility of double-counting two fragments of a single strike. For comparison, the longest documented fulgurite yet discovered has a length of 5 meters^1^, and hence this single individual is about a third of the total length of all of the fulgurites recovered in the present study. The median length of the fulgurites from these sand mines in the present collection is about 5 cm, with no significant variations in diameter across these lengths. As a result, the systematic collection of fulgurites might yield distributions where length is proportional to the abundance of each fulgurite of a specific width.

Alternatively, if we treat each fulgurite as a separate event, then there exists the possibility that a single event may be counted multiple times if the fulgurite fragmented before collection. However, if multiple events have the same energy, then counting by numerical frequency considers this multiplicity. Regardless, both approaches give very similar slopes between log E (MJ/m) and frequency/cumulative length (Table 1), and the median (50%) energy associated with fulgurite formation in both cases is about 1.4 MJ/m, and the standard deviation is ×/÷ 3.

The total energy recorded within the fulgurite tube can also be approximated by multiplying the energy per unit length by the length of a given fulgurite. Such a calculation assumes that breakage of the glass was minimal since formation. This scenario is presented for the sake of completeness, but given the fragile nature of fulgurites, is likely inaccurate. However, since most of the fulgurite fragments have similar lengths, the energy distribution is also lognormal, like the energy per unit length distributions. The regression line fit to the lognormal total energy vs. frequency distribution is the strongest of these calculations (R^2^ = 0.9852), though the slope differs from the prior two approaches.

Comparison between the fulgurite distributions and between the simulations (SI) suggests that, as long as the fulgurite fragments are collected without bias, and as long as the fulgurites have experienced roughly the same fracturing history, then the fulgurite distributions are most consistent with being lognormal with respect to energy per length. Consequently, the resulting distribution of total energy should also be lognormal even when fractured, as is borne out by the above data.

The density of a subset of the fulgurites subset is found to be 1.8 ± 0.1 g/cm^3^ (1**σ**). The density of the accompanying sand is 1.7 g/cm^3^, and of the individual quartz grains that make up the sand 2.6 g/cm^3^.

## Discussion

The relationship between the energy *E* per unit length of a given lightning strike and frequency of that given strike having energy *E* or more follows a lognormal distribution. Many lightning parameters follow a log-normal distribution with respect to the frequency of that parameter occurring, including flash duration, voltage, charge transferred, stroke interval, current duration and action integral^13^,^14^,^15^. The slopes of the log E (MJ/m) plots vs. frequency or cumulative length (normalized to probability 1) are both close to −0.6. Intriguingly, the slope of frequency vs. charge or impulse peak current measured by Pichler *et al*.^14^ also bear slopes of −0.6, nearly identical to these fulgurite morphology slopes^14^.

For a poorly conductive target material that does not vary significantly in composition in aerial extent, the electric field must exceed the breakdown field strength of the material to propagate a spark. For sand, this strength is less than 8 MV/m, the breakdown strength of quartz. Air has a lower breakdown strength (~1 MV/m at 1 sea level)^16^, hence the breakdown strength of sand should be less than quartz alone. Thus the energy per unit length deposited by lightning into the sand should be a function of the number of electrons that propagate through the sand as the insulating capability of the sand fails, in addition to the amount of thermal energy transferred by the column of heated plasma adjacent to the fulgurite. The amount of charge transferred by lightning is known to follow a lognormal distribution function^14^, and hence the energy deposited by lightning might be expected to follow the same. The lognormal nature of charge transferred may hence be the source of lognormal distribution of fulgurite energy per unit length, though other parameters may also play a role.

### Accuracy of “fossilization” of energy per unit length in fulgurites

The density of the fulgurites appears to have not changed substantially from the quartz sand during heating. This implies that there was little movement of the molten silica during the lightning strike. If the column of heated air had pushed the molten glass radially outward by the expansion of gas, the glass would have increased in density to arrive at a value closer to the density of silica glass (~2.6 g/cm^3^) as air was displaced from pore spaces. Instead, the similarity in density between sand and glass suggests the glass is as porous as the sand. The increase of about 0.1 g/cm^3^ may be attributed to the presence of unmelted grains of quartz captured on the exterior of the fulgurite. These grains, when frozen onto the surface of the fulgurite, will increase the density of the fulgurite, especially if the size of the sand grains is not significantly smaller than the size of the fulgurite wall. Given the sand grains range from 0.1 to 0.5 mm, and the fulgurite glass walls are commonly about 1 mm thick, the embedding of quartz grains alone would give an increase in density from sand. Thus, we conclude that the fulgurites capture the original energy of the lightning without significant modification or post-lightning fluid movement.

### Distribution residuals

We postulate that the energy vs. frequency reported in Fig. 3 may record up to four separate phenomena. The data residuals with respect to the calculated regression line demonstrate three points where discontinuities occur (Fig. 4). These deviations occur for all three lognormal plots. One of these occurs at the high-energy regime (6 MJ/m and larger, or 0.2 MJ total energy). This group constitutes about 3–4% of all fulgurites, and we speculate that this discontinuity occurs when the lightning that forms the fulgurites becomes positive. Positive lightning strikes have a higher mean energy than negative lightning strikes, and are known to comprise about 4% of all strikes annually^17^,^18^. The lower energy per unit length (<6 MJ/m) likely represents fulgurites formed by negative lightning strikes, by far the most common type of cloud-to-ground lightning event. The discontinuity in frequency that occurs from 1 to 6 MJ/m (0.08 to 0.2 MJ) may hence be due to a mixing of two different lightning types with differing distributions in lognormal space: a high energy variety that is tapering off as the energy decreases, and a low energy variety that becomes more important at lower energies. Alternatively, these deviations are also demonstrated in the fulgurite distribution simulations, and result from the stronger nature of thicker fulgurite fragments. If bigger fulgurites don’t fragment as easily as smaller fulgurites, then these changes to slope are expected. From 1 MJ/m to below 0.3 MJ/m (or 0.005 to 0.08 MJ total energy), the residuals are roughly constant, implying a match to the calculated regression slope in lognormal space. Finally, below 0.3 MJ/m (0.005 MJ total energy) are the lowest energy fulgurites. These probably deviate from the trend as fulgurites that are very thin are also more fragile, are smaller, and are more difficult to recover, hence the accurate collection of fulgurites within this size range is difficult. Proof of any of these points would require characterization of specific lightning strikes followed by observation of the morphology of fulgurites formed.

### Energy dissipated to form fulgurites

The average energy required to form these fulgurites in quartz sand is about 1.4 MJ/m, with a standard deviation of ×/ ÷ 3. In contrast, the energy per unit length of lightning traveling through air is between 10^3^ and 10^4^ J/m^19^. If the total energies along these lengths are considered, a 5 km lightning strike through air dissipates about 5 MJ, and a corresponding 10 cm fulgurite dissipates only about 0.1 MJ. The energy estimates^20^ of Maggio *et al*. also suggest lightning strike energies of 10^8^–10^9^ J, implying 1% or less of the energy of lightning is captured in fulgurite formation. However, within the ground material in which a fulgurite forms, the energy per unit length is much higher than the energy per unit length in the atmosphere by about a factor of 100–1000. This is likely a result of the higher density of the target material (1700 kg/m^3^) compared to the atmosphere (~1 kg/m^3^), which also vary by a factor of 1000. Thus the energy dissipation by lightning is a function of the density of the material through which it travels.

## Conclusions

We have reported here the first attempt to determine the lightning energy distribution from fulgurites, and one of the first datasets of cloud-to-ground lightning energy delivery measured as a function of damage to the solid earth surface. The frequency distribution of the energy required to form a fulgurite follows a lognormal relationship, consistent with many lightning phenomena. Lognormal relationships are a natural consequence of the multiplication of normal probabilistic distributions and imply the energy transferred to the ground, like other lightning phenomena, is skewed towards high energy events with higher frequency than a typical normal distribution.

The current work presents a new approach to determining lightning parameters. Measurements of these lightning parameters are typically done either by analyzing lightning initiated by launching rockets^21^ or by analysis of strikes to towers and tall buildings^22^. These methods have provided most of the parameters of lightning as it relates to these discharges striking a conductive target. The work here provides a means of directly determining energy as a lightning parameter in cloud-to-ground strikes on an insulating material (fine quartz sand). With an average energy of 1.4 MJ/m in conductive materials, and a peak of >20 MJ/m, this data may be of use in anticipating lightning damage from cloud-to-ground strikes. If fulgurites occurring in other materials, such as clays or rock, could be investigated in a fashion similar to the present study, better resolution on the energy of lightning may be possible as clay and rock fulgurites are usually better at preserving the thermal effects resulting from lightning^6^.

## Methods

### Locality

Fulgurites from two sand mines, Pit #1 and Pit #4 operated by C. C. Calhoun, Inc., in central peninsular Polk County, Florida (USA) were chosen for this analysis (Fig. 2). These mines are located along, and near, the crest of the Lake Wales Ridge, in Polk County, Florida. Up to 20 meters of siliciclastic sediments, mapped by Campbell as the Cypresshead Formation^23^,^24^,^25^, are exposed in the pits. These sands consist of at least 98% SiO_2_ with the remainder clay and oxide minerals, and have similar grain size distributions. Materials from the Cypresshead Formation were transported from Georgia into Florida by longshore currents^26^ and deposited in the Late Pliocene to Early Pleistocene^27^. Depositional environments have been interpreted as nearshore marine to brackish^26^,^28^,^29^.

**Figure 1:**
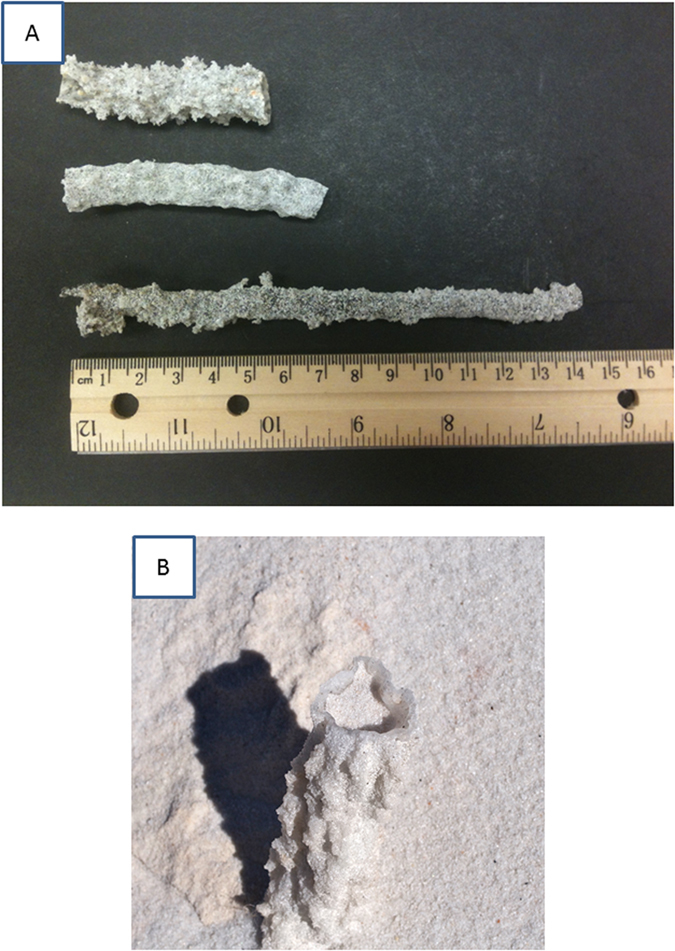
(**A**) Exemplary fulgurites collected from the field area. Surficial differences likely result from different initial physical conditions (e.g., percent water in sand). Color differences are apparently due difference between the thicknesses of the glass wall, which are 1.1 ± 0.1, 0.9 ± 0.3, and 1.6 ± 0.3 mm from top to bottom (taken from 10 measurements, with 1 standard deviation given as the error). (**B**) Fulgurite found *in situ*. Diameter is about 1.2 cm.

**Figure 2:**
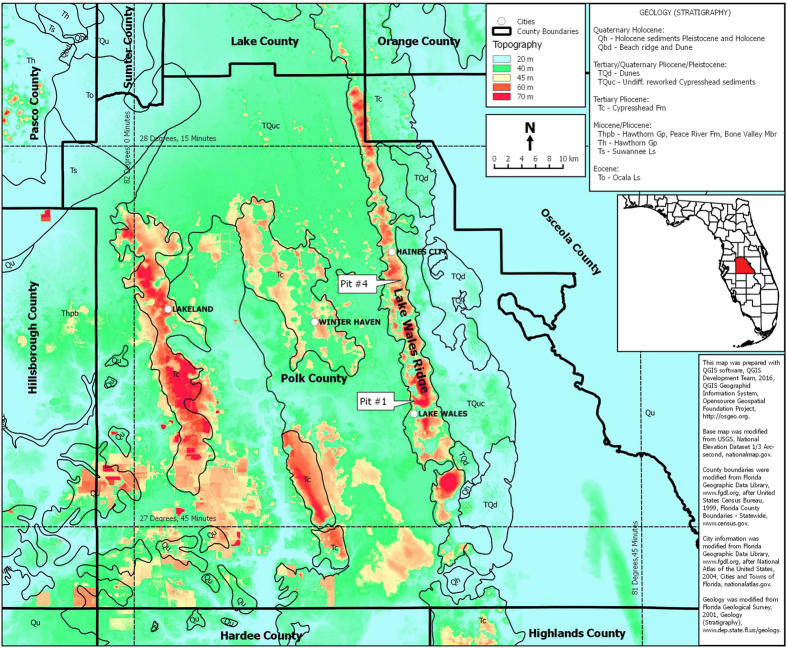
Polk County, Florida, USA digital elevation map showing sample localities for fulgurites. This map was prepared with QGIS software (QGIS Development team, 2016, http://osgeo.org). Base map was modified from USGS National Elevation Dataset 1/3 arc-second, (www.nationalmap.gov). County boundaries were modified from the Florida Geographic Data Library (www.fgdl.org) after the United State Census Bureau, 1999, Florida County Boundaries – Statewide (www.census.gov). City information was modified from the Florida Geographic Data Library (www.fgdl.org) after the National Atlas of the United States, 2004, Cities and Towns of Florida (www.nationalatlas.gov).

**Figure 3:**
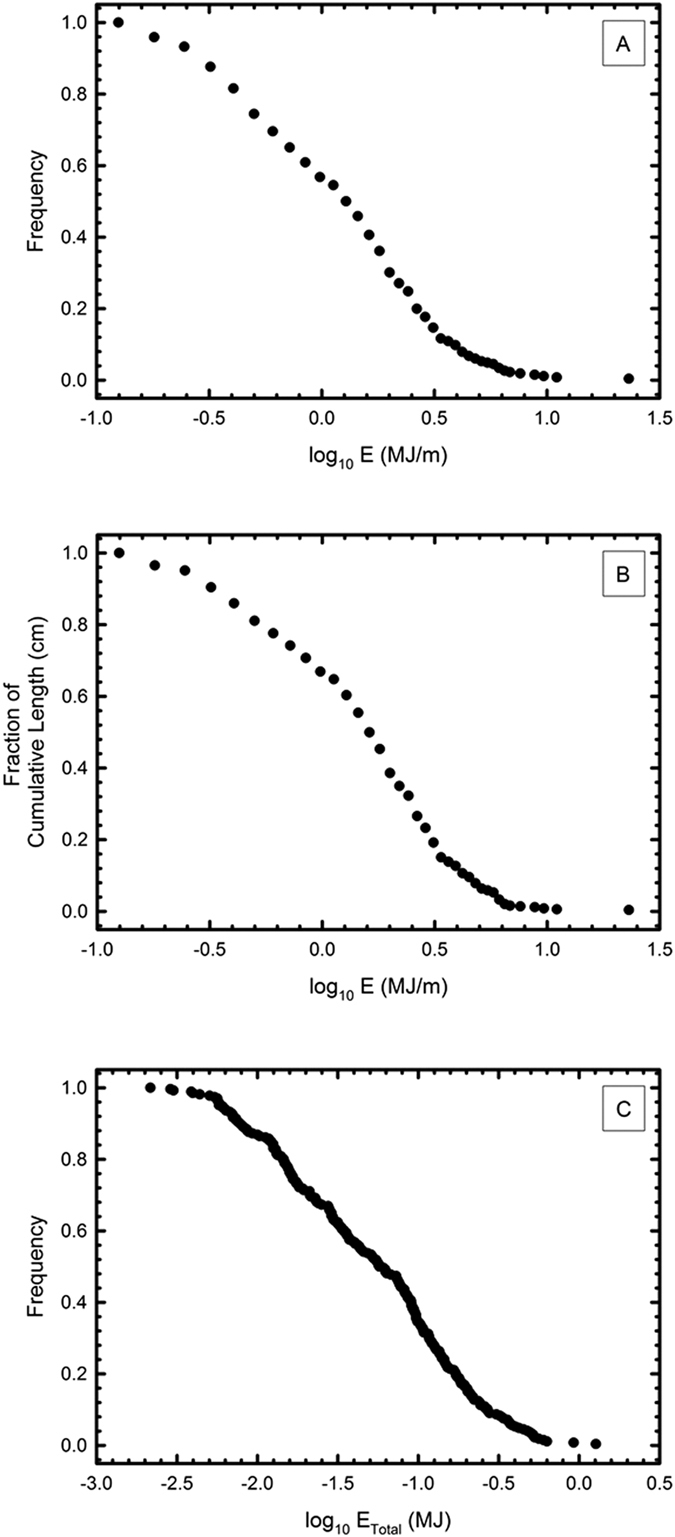
Lognormal frequency distributions. (**A**) Distribution of energy per length (MJ/m) vs. number. Each fulgurite, is assigned a number corresponding to the number of fulgurites equal to or larger than it with respect to energy per unit length. These numbers are then divided by the total number of fulgurites collected (266). (**B**) Distribution of energy per length (MJ/m) vs. cumulative length. As above, each fulgurite is arranged in descending energy per unit length. The energy is then graphed against the cumulative length of each fulgurite bearing that energy or more. This length is then divided by the total length (14.659 m). (**C**) Distribution of total energy (MJ) vs. number. The total energy is obtained by multiplying the energy per unit length determined from the diameter of the fulgurite by its length. Fulgurites are then arranged in order of decreasing total energy, and each fulgurite is given a number based on the number of fulgurites equal to or greater than the calculated total energy. These numbers are then divided by 266, the total number of fulgurites collected.

**Figure 4:**
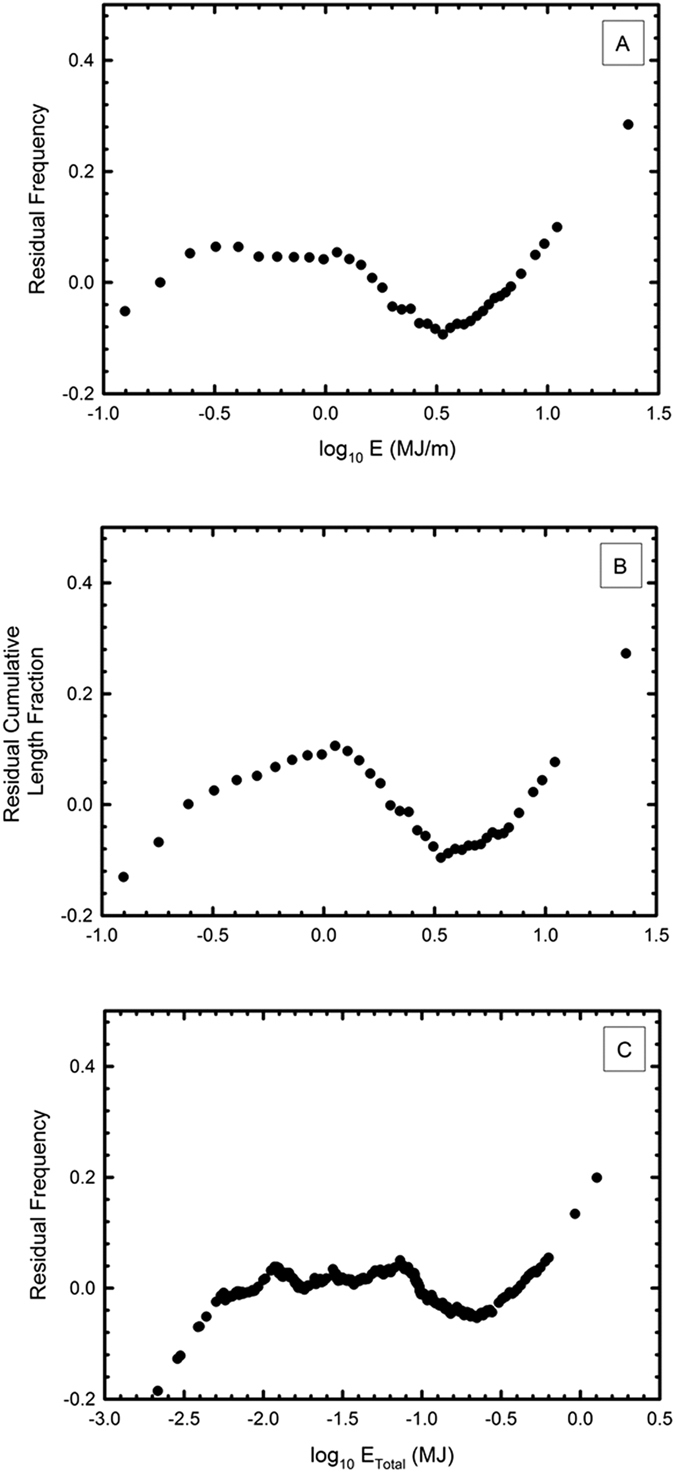
The calculated residual for each data point (distance between the calculation regression line and the actual data point with respect to y-axis) for (**A**) log E (MJ/m) vs frequency, (**B**) log E (MJ/m) vs cumulative length, and (**C**) log E (MJ) vs frequency.

Fulgurites found at these mines, and others in the region are most abundant in clean, white, fine-to-medium-grained quartz sand (~30–35% porosity). We suspect the white sand unit to be aeolian (wind-deposited) in origin: at Pit #1 the unit consists of a single set of large-scale, high-angle, cross-beds measuring about 10 meters thick. The unit is somewhat thinner (about 7 m) at Pit #4; and bedding is massive, exhibiting little sedimentary depositional features. These characteristics suggest that sand was deposited as a dune and has been above sea level for a long time. A total of 266 fulgurites were collected from these mines, for a cumulative length of over 14 meters (Fig. 1). Note that fulgurites with incomplete cylinders are not considered in this study, as it is difficult to estimate the diameter and correct length of broken fulgurites. No branching fulgurites—fulgurites with bifurcations—were collected amongst these fulgurites; fulgurites forming in sand usually are unbranched^6^. Fulgurites, when found *in situ*, were oriented vertically, and had no indication of radial outward development. Every fulgurite we observed within the field area (~0.02 km^2^) was collected, and the internal void diameters (measured four times, twice at the top of each fulgurite and again at the bottom) and total fulgurite lengths were measured and recorded in centimeters. Since complete fulgurites are difficult to collect as these objects are prone to fracturing, for most of these fulgurites we do not have the full length. Additionally, the sand mines are still actively producing sand as a construction material hence heavy equipment crushes and distributes fulgurite fragments across the surface of the mine.

The process of mining inhibits the collection of complete fulgurites as the glasses are frequently cleaved during excavation. Fulgurites that are excavated during mining are separated from the sand and usually end up in waste spoil piles; fulgurites from these locations were not collected. The remainder of the fulgurites exposed to the surface erode out, frequently fracturing and becoming further distributed to the surface. Although it would be ideal to collect and excavate all of the complete fulgurites within a sand mine, this is not feasible financially. However, mining typically cuts through sand in ~1 m high layers (the height of the excavator). To this end, fulgurites that are cleaved and erode out represent a cross-section of the lightning that has struck over about a 1 m region in height within these dunes as the mining proceeds through the sand.

### Sand and Fulgurite Composition

The sand mines here are characterized by being composed of almost exclusively quartz sand, and similar mines in the region are over 99.2% SiO_2_^30^. Quartz is the only mineral identified in X-ray diffractograms of powdered samples of the sand, and Raman analysis of the sand also only shows quartz (see SI). Raman data from a subset of five of these fulgurites only detected SiO_2_ phases, principally quartz and lechatelierite glass (SI)^4^. Additionally, a single ICP-MS analysis of a fulgurite had ~99% SiO_2_ (SI). Consequently, the calculations done here assume the mineralogy of the sand consists exclusively of quartz.

These findings are consistent with prior work done on sand fulgurites. The fulgurites found here would be considered to be type I fulgurites according to the scheme of Pasek *et al*. (2012)^6^. Other studies of type I fulgurites have shown that these objects are typically composed of >98% SiO_2_^6^,^31^,^32^.

### Calculations

The energy required to vaporize quartz as SiO_2_ from room temperature is calculated using HSC Chemistry, a thermodynamic equilibrium modeling code that has been previously used to determine the Solar system chemistry of sulfur^33^, the composition of Europa’s subsurface ocean^34^, and fulgurite formation processes^6^. This model determines the energy at one bar of pressure required to promote the reactions:


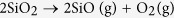



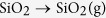


where the vaporization of SiO_2_ is accompanied by the decomposition of this material to SiO_2_, SiO and O_2_ gases. The code determined the vaporization of SiO_2_ approximately 0.94 MJ per mole (~15.7 MJ/kg,). Correspondingly, the relationship between fulgurite internal diameter and energy per unit length (*E*) required to vaporize the material to make a fulgurite is equal to:


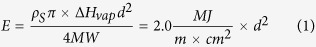


where *ρ*_*S*_ is the density of sand (here at ~1.65 g/cm^3^), Δ*H*_*vap*_ is the energy required to vaporize SiO_2_ from room temperature (the above 0.94 MJ/mole), *MW* is the molecular weight of SiO_2_ (60.08 g/mole), and *d* is the internal diameter of the fulgurite in centimeters. We do not consider further energy transfer in the formation of the glass wall (e.g., the melting of the sand to form the fulgurite, and heating of adjacent sand) that may store up to 20% of the energy of a lightning strike, and the heating and ionization of the gas column within the fulgurite is not considered. These data cannot be readily determined from the fulgurites collected. These energy estimates are necessarily minima, and the actual energy transfer may be more than 25% of our calculated values.

We also omit the enthalpy of vaporization of water from these calculations (40 kJ/mol). This omission is based on two factors: 1) given that the geologic setting of these sand dunes is consistent with being above sea level since their deposition^35^, it is reasonable to assume that the conducting subsurface aquifer is suitably distant from the surface of the sand dunes such that the fulgurites occurring within the sand were not saturated with water. Water saturation requires about 2.5 cm of rainfall to saturate 5 cm of sand, and 5 cm being the median fulgurite fragment length found here. While a rainfall of 2.5 cm in a single storm event is not uncommon in Florida, most lightning precedes the heaviest rainfall^36^. 2) The sand has a porosity that allows about one mole of water per mole of sand, and the exclusion of 40 kJ/mol (vs. 940 kJ/mol) from these calculations should not introduce significant error (about 0.02 log units in MJ/m). However, the surfaces of individual fulgurites range from smooth to wrinkled to covered in fractal-like branching patterns (Fig. 1). These differences are likely attributable to differences in the physical conditions at the time of formation, including water content. Additionally, differences in fulgurite glass thickness may be partially attributed to sand water content, as wetter sand should transfer heat more readily than dry sand. Thus, thicker-walled fulgurites may be more common in wet sand.

In addition to measuring the shape of fulgurite cylinders, we also determined the density of a subset (n = 12) of the glasses in fulgurites using Archimedes’ displacement of water technique to determine volume. The fulgurites chosen were completely open tubes, so that a measurement of the density of the glass could be performed without also measuring gas that might have been pinched in by the collapse of the tube. The density of the accompanying sand was also measured by both a mass per unit volume technique, and by the Archimedes displacement principle. The latter was used specifically to estimate the bulk density of the quartz grains.

### Model Distributions

Since we do not presume to have the complete length of most of the fulgurites within our collection as nearly all were discovered exposed on the sand surface, we performed a set of calculations to determine how the measured collection compares to a simulated distribution.

We modeled the distribution of fulgurite fragments as a function of energy per meter vs. cumulative length and vs. cumulative number, and total energy vs. cumulative number to determine the resulting distributions (see SI). These models tested six scenarios: energy per length distributions that were normal or lognormal, and length distributions that were constant across all energy per length distributions (in other words, one meter fulgurites were formed at the lowest energy per unit length and at the highest energy), or length distributions that were normal or lognormal. Each fulgurite was assigned a specific energy, and a specific length.

To these distributions we applied a “fracture event” that broke each simulated fulgurite into smaller, equal parts. This fracture event assumed the following: since the weakest point of a fulgurite will be at the midpoint of its length, each fulgurite was assumed to fracture in half, and the fragments may then fracture subsequently in half. A fulgurite will fracture as many times as possible until it reaches a specific length, which we establish empirically (SI). The diameter of the fulgurites is weakly correlated with length (R = 0.38), which establishes the maximum length allowed in the simulation. Thicker fulgurites should be expected to be somewhat stronger, and hence produce longer lengths. From these fragments and energy per unit length distributions, we compare our simulated distributions with those from the actual suite of fulgurites.

## Additional Information

**How to cite this article**: Pasek, M. A. and Hurst, M. A Fossilized Energy Distribution of Lightning. *Sci. Rep*. **6**, 30586; doi: 10.1038/srep30586 (2016).

## Supplementary Material

Supplementary Information

## Figures and Tables

**Table 1:** Linear regression line slope and y-intercept for lightning energy frequencies.

X	Y	m	b	R^2^	Value at 50%
Log E (MJ/m)	f (total = 1)	−0.5877	0.5211	0.9496	1.3 MJ/m
Log E (MJ/m)	length (1466 cm = 1)	−0.6174	0.5731	0.9438	1.6 MJ/m
Log E (MJ)	f (total = 1)	−0.4986	−0.1435	0.9852	0.051 MJ
