# Supplementary material for: A Fossilized Energy Distribution of Lightning
Source: Sci Rep. 2016 Jul 28;6:30586. doi: 10.1038/srep30586 (PMC4964350; doi:10.1038/srep30586)
Supplement: Supplementary Information [file srep30586-s1.pdf]

A fossilized energy distribution of lightning: supplementary information

Matthew A. Pasek[1] and Marc Hurst[2]

[1]School of Geoscience, NES 204, University of South Florida, 4202 E Fowler Ave, Tampa FL 33620 mpasek@usf.edu, Phone: 813-974-8979

[2]Independent Geological Services, Inc., 4432 Burlington Drive, Winter Haven, FL 33880, marc.hurst@independentgeo.com

Correspondence and requests for materials should be sent to M. A. P. (mpasek@usf.edu)

ABSTRACT

Here we provide the compositional information on the sand and fulgurites (XRD, Raman, ICP-MS), and details on the simulations of fuglurite distributions with consideration of fracturing.

Model details. A simple set of models were developed to see if the fulgurite energy per length and total energy distributions observed could be consistent with varied starting assumptions. These assumptions were as follows. 1) The energy per length follows a normal distribution, assuming an average of 1 MJ/m, and a standard deviation of 0.3 MJ/m. 2) The energy per length follows a lognormal distribution, assuming an average of 1 MJ/m, and a standard deviation of a factor of 2.8, consistent with the measured values in the fulgurites. 3) The starting length of each fulgurite was assumed to be a constant 1 m. 4) The starting length of each fulgurite was assumed to follow a normal distribution with an average length of 1 m and a standard deviation of 0.3 m. 5) The starting length of each fulgurite was assumed to follow a lognormal distribution with an average length of 1 m, and a standard deviation of a factor of 2.8 (identical to the lognormal energy per length distribution). This gives a total of six scenarios (varied MJ/m and length L).

Each scenario had 20 different model fulgurites. Each fulgurite began with a specific energy per unit length (correlating to diameter) and a specific total length. For each of these, the distribution between cumulative length and energy per length (MJ/m) could be calculated over ±3.15 standard deviations. The model fulgurite energy and length was calculated every third of a standard deviation, starting with the highest energy fulgurites and summing the length from these of each lower energy fulgurite.

To these fulgurites we applied a simple fracturing model. This model, which was derived from the diameter-length correlation of the fulgurites (Figure S1)

Max length (cm) = 2.3105 x diameter (cm) + 3.613

provides an artificial maximum length of each fulgurite fragment. If the fulgurite or fragments were longer than this maximum length, the fulgurite was assumed to break in two, yielding two smaller fragments. Note that this “max length” is better described as an average length for these fulgurites, but will be used in the current model as a maximum to provide the number of fragments expected for each fulgurite. We acknowledge this to be a simplification of the actual fracturing process.

Each fulgurite will generate a total of

$$fragments = 2^{log_2 \frac{length}{\max length}}$$

where the *length* over *max length* was rounded up or set to 1 if the *length* was smaller than the *max length*. Thus each fulgurite is assumed to generate a specific number of fragments that is a multiple of 2. With this data, a cumulative number vs. energy per length could be constructed.

Finally, the energy per unit length is multiplied by the fragment length to give a total energy preserved within each fragment. The energy vs. cumulative number could then be constructed.

The case of energy per length following a normal distribution with length being constant (scenario 1 + 3 above) can be considered as an example. A normal distribution of lightning energy per unit length with an average of 1 MJ/m and 0.3 MJ/m standard deviation ranges from 0.05 MJ/m to 1.95 MJ/m over 3.15 standard deviations. Each of these fulgurites is then assigned a length, which in this case is 1 m. Thus

the fulgurite with 1.95 MJ/m is the beginning point of the distribution for cumulative length vs. MJ/m. The second point is 1.85 MJ/m, and to organize this data into a cumulative length distribution diagram, its length is added to the preceding fulgurite's length. This is done sequentially to reach the final fulgurite, 0.05 MJ/m, which provides the final 20m of length.

Subsequently, the max length of each of the 20 fulgurites is determined using the empirical relationship above, and the number of (equivalent) fragments is determined from the initial 1 m of length. For the 0.05 MJ/m fulgurite (0.025 cm diameter), we expect each fragment to be smaller than 3.7 cm, and hence there will be 32 fragments each 3.125 cm long. A 1.95 MJ/m fulgurite will have a maximum length of 5.9 cm, and hence there will also be 32 fragments each 3.125 cm long (as opposed to 16 fragments 6.25 cm long). Each fulgurite hence provides 32 fragments in this scenario, and the corresponding distribution is akin to the cumulative length distribution.

Finally, by multiplying each fulgurite fragment's length by its energy per length, a total energy preserved in each fragment can be determined. Comparing these total energies to the cumulative number yields the distribution provided as Figure S2-F.

These results of these simulations can then be compared to the fulgurite data (Figure S2).

Compositional Data.

A sample XRD of the quartz sand is provided as Figure S3. This diffractogram was acquired on a BTX Olympus desktop XRD for 2 hours (400 scans), and is comparable to XRD patterns from quartz from King County, Washington, USA, from the rruff.info website (http://rruff.info/quartz/R040031).[1]

Raman of both the quartz sand and of the fulgurite demonstrated only SiO_2 and lechatelierite glass. Example Raman spectra of fulgurite quartz, lechatelierite, and the rruff.info quartz sample are provided as Figure S4.[1] These Raman data were acquired on an EnWave μSense Raman microscope over 30 seconds scanning time with a 785 nm laser.

Additionally, a single fulgurite was crushed and analyzed by ICP-MS (a ThermoFinnegan Element2 ICP-MS) following established techniques (e.g., Haynes et al. 2010).[2] These data show almost pure SiO_2 (Table S1).

Figure S1. Relationship between diameter and length of the 266 fulgurites.

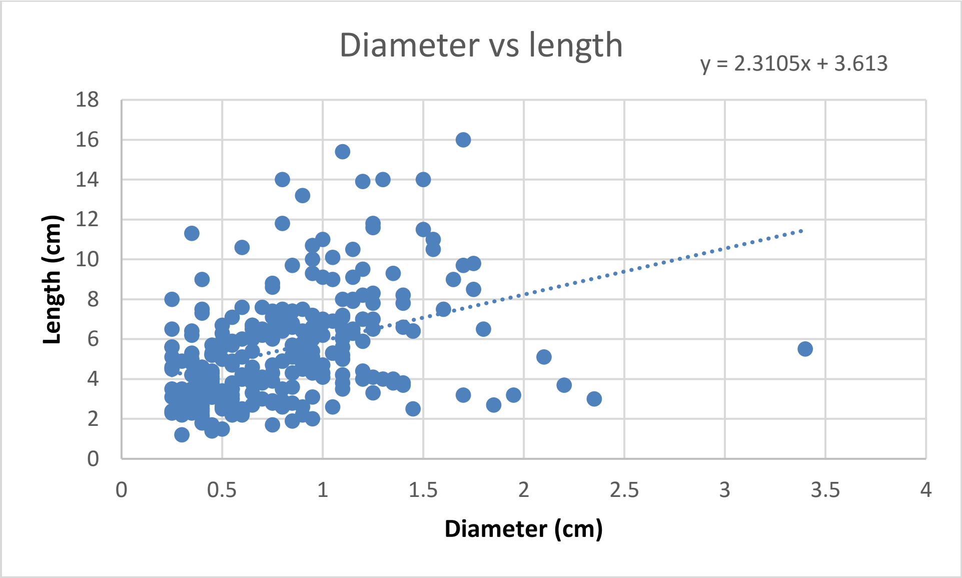

Figure S2. Comparison between fulgurites and modeled fulgurite distributions. A) Fulgurite energy per unit length vs. cumulative length (identical to Figure 3B but with a linear scale). B) Fulgurite energy per unit length vs. cumulative number (identical to Figure 3A but with a linear scale). C) Fulgurite energy vs. cumulative number (identical to Figure 3C but with a linear scale). D) Modeled fulgurite distribution in terms of energy per unit length vs. cumulative length assuming a normal distribution of energy per length, and all fulgurites forming with constant length. E) As D), but plotting energy per length against cumulative number, after applying fractures. F) As D), but plotting energy preserve per fragment against cumulative number. G)-I) Y-axes are the same as the graphs vertically above. Modeled distributions are based here on energy per unit length following a normal distribution, and length following a normal distribution (the average energies correspond to the longest lengths). J)-L) As before, but assuming a lognormal distribution, with the highest energy per length corresponding to the longest length. M)-O) Modeled distribution assuming a lognormal energy distribution and a constant fulgurite length on formation (each fulgurite is 1 m). P)-R) As in M)-O), but assuming a normal distribution with respect to length. S)-U) As in M)-O) but assuming a lognormal distribution with respect to length.

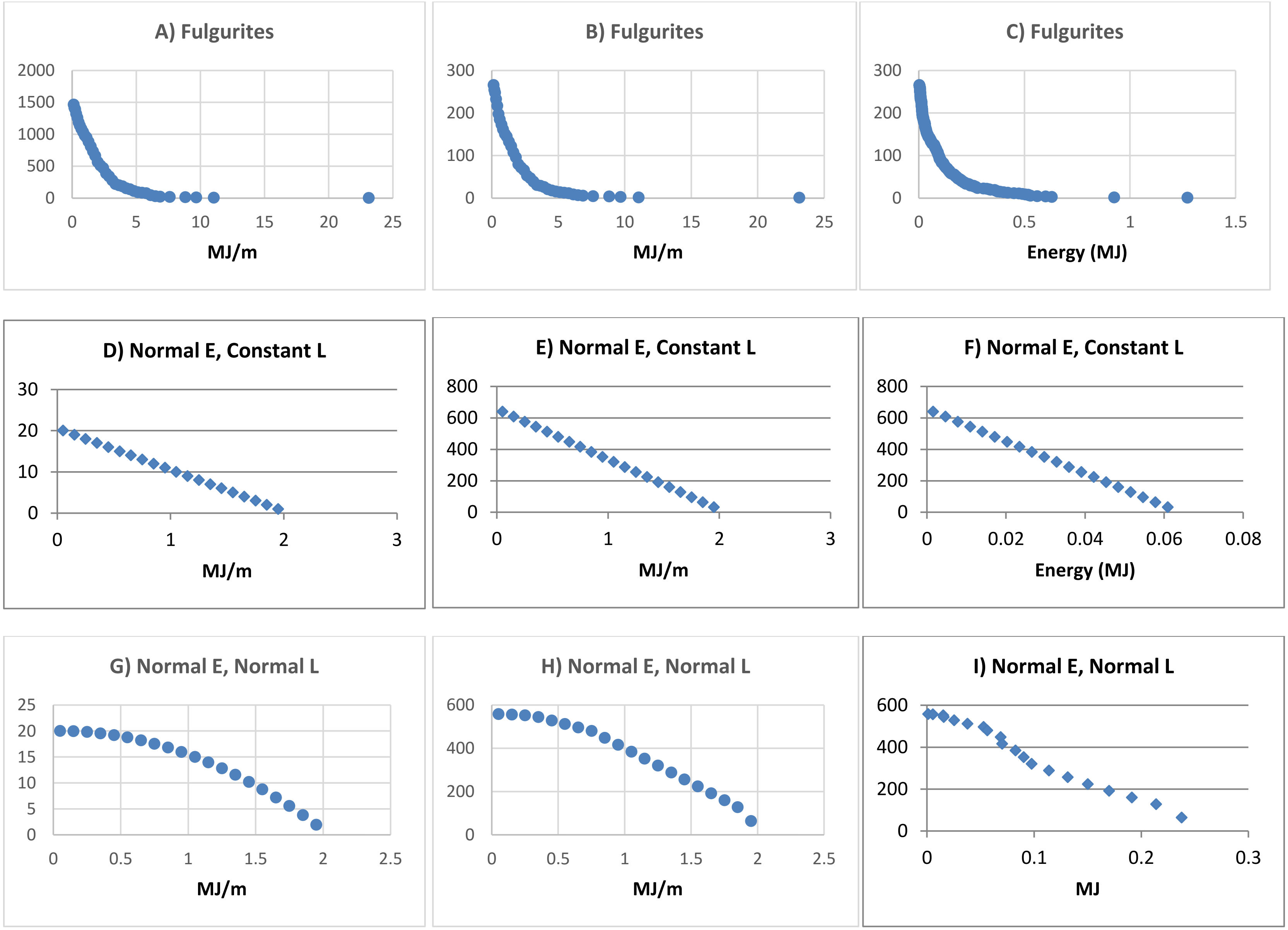
A) Fulgurites
MJ/m
B) Fulgurites
MJ/m
C) Fulgurites
Energy (MJ)
D) Normal E, Constant L
MJ/m
E) Normal E, Constant L
MJ/m
F) Normal E, Constant L
Energy (MJ)
G) Normal E, Normal L
MJ/m
H) Normal E, Normal L
MJ/m
I) Normal E, Normal L
MJ

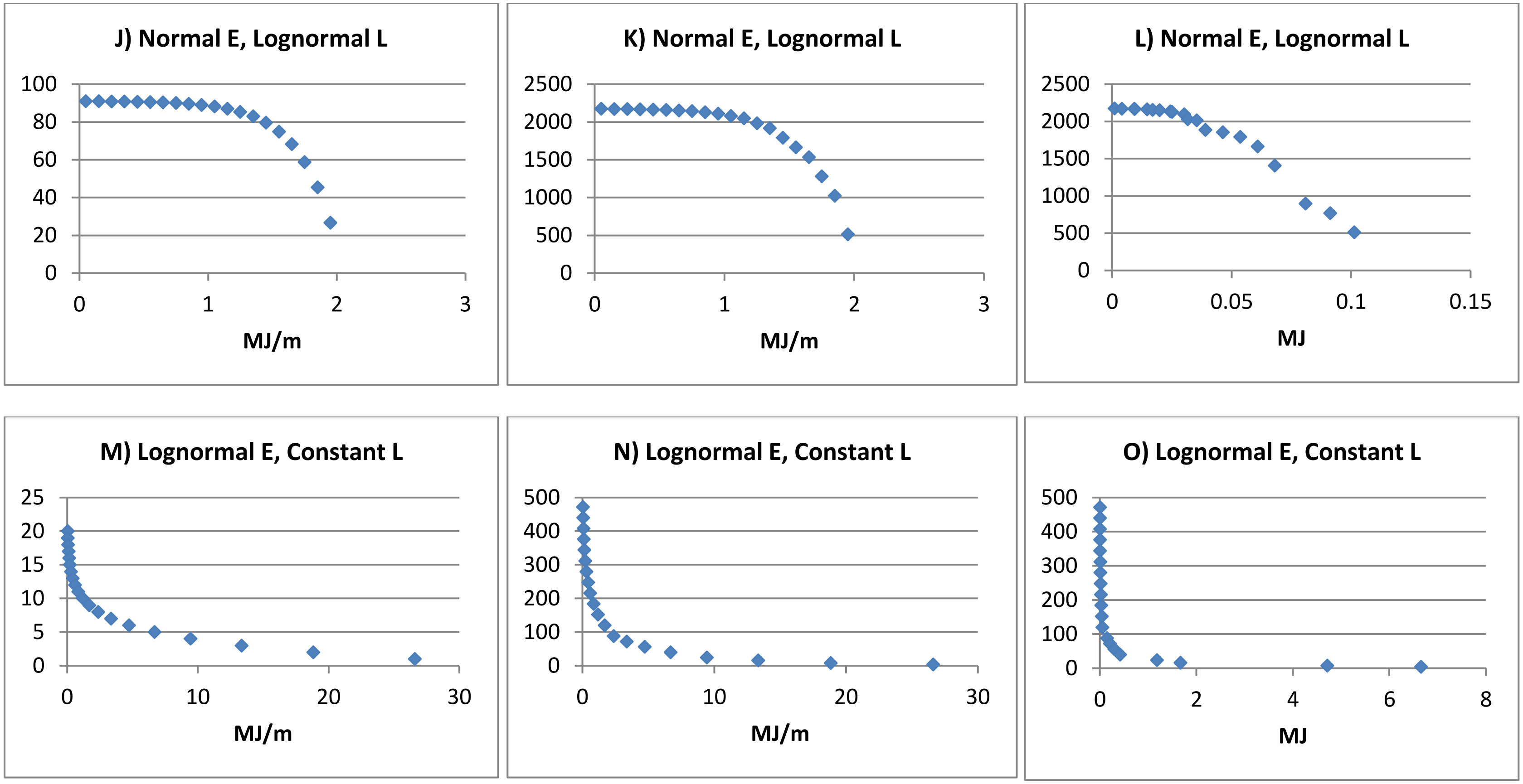
J) Normal E, Lognormal L
MJ/m
K) Normal E, Lognormal L
MJ/m
L) Normal E, Lognormal L
MJ
M) Lognormal E, Constant L
MJ/m
N) Lognormal E, Constant L
MJ/m
O) Lognormal E, Constant L
MJ

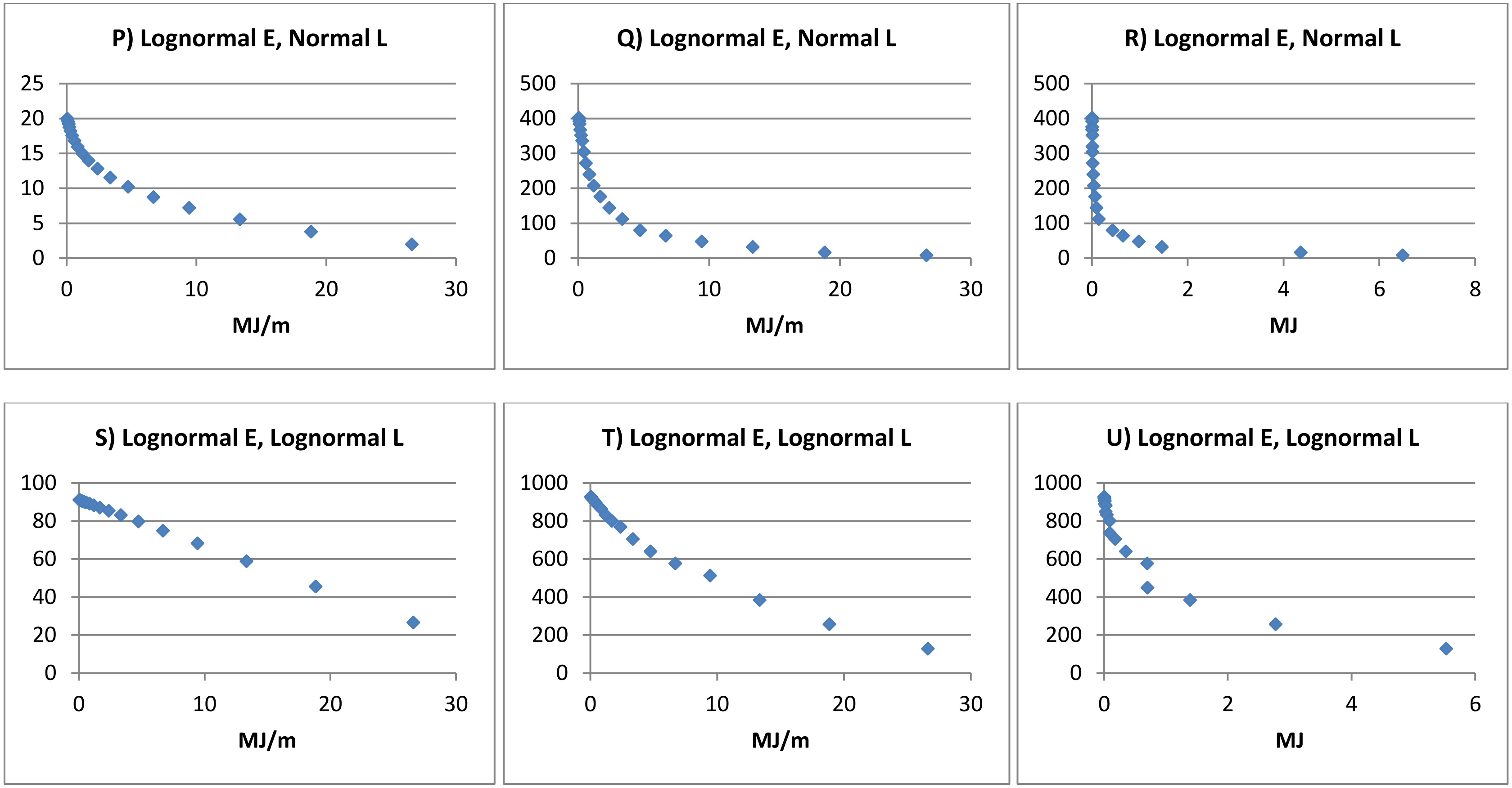
P) Lognormal E, Normal L
MJ/m
Q) Lognormal E, Normal L
MJ/m
R) Lognormal E, Normal L
MJ
S) Lognormal E, Lognormal L
MJ/m
T) Lognormal E, Lognormal L
MJ/m
U) Lognormal E, Lognormal L
MJ

Figure S3. X-ray diffractograms of the A) bulk sand from the Polk County mines compared to B) samples of quartz from King County, Washington USA. Y-axis is arbitrary intensity units.

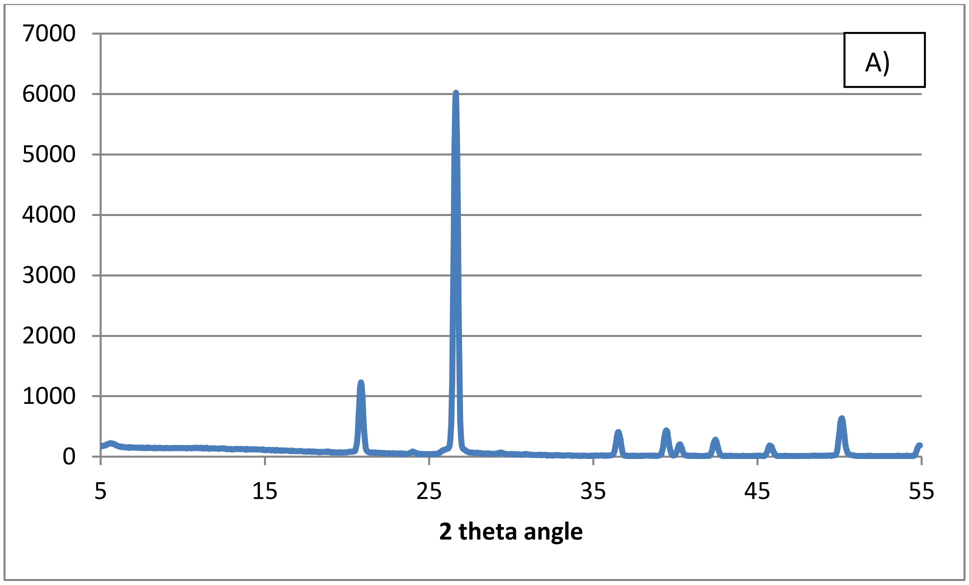

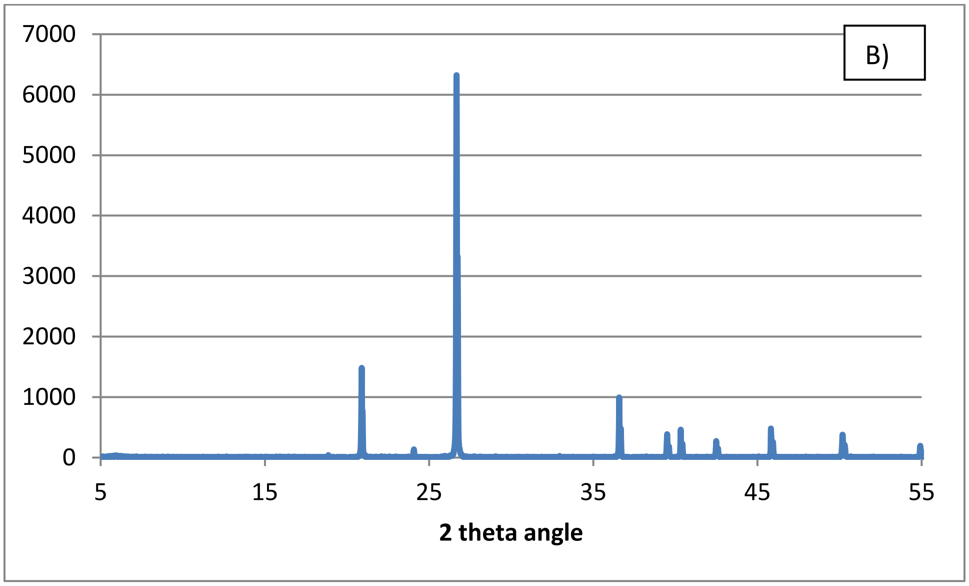

Figure S4. Sample Raman spectra of Polk County fulgurite surfaces showing A) quartz, with a comparison to B) authentic quartz from rruff.info website. C) provides the spectrum of the fulgurite lechatelierite (glass of composition SiO_2) and should be compared with Carter et al. (2010).[3] Y-axis is arbitrary intensity units.

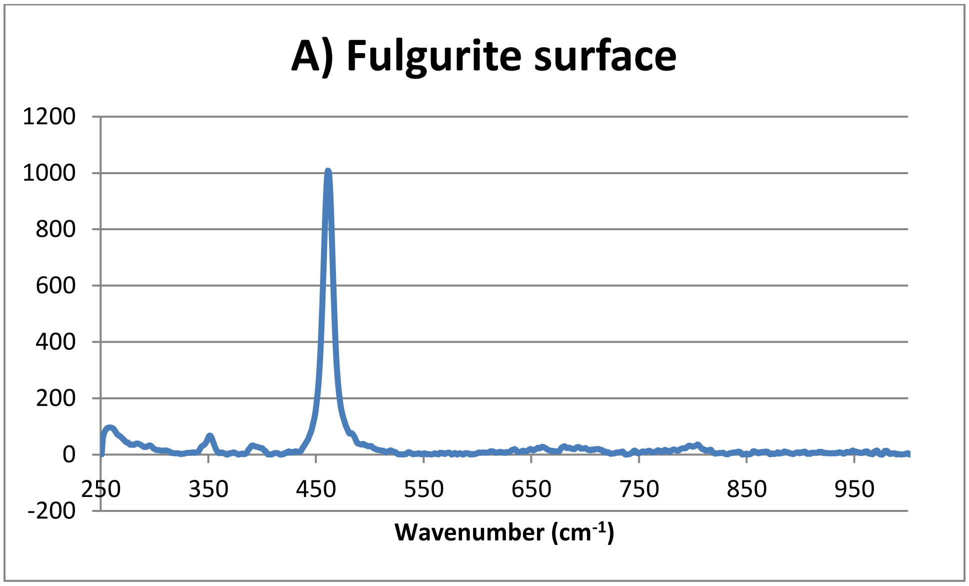

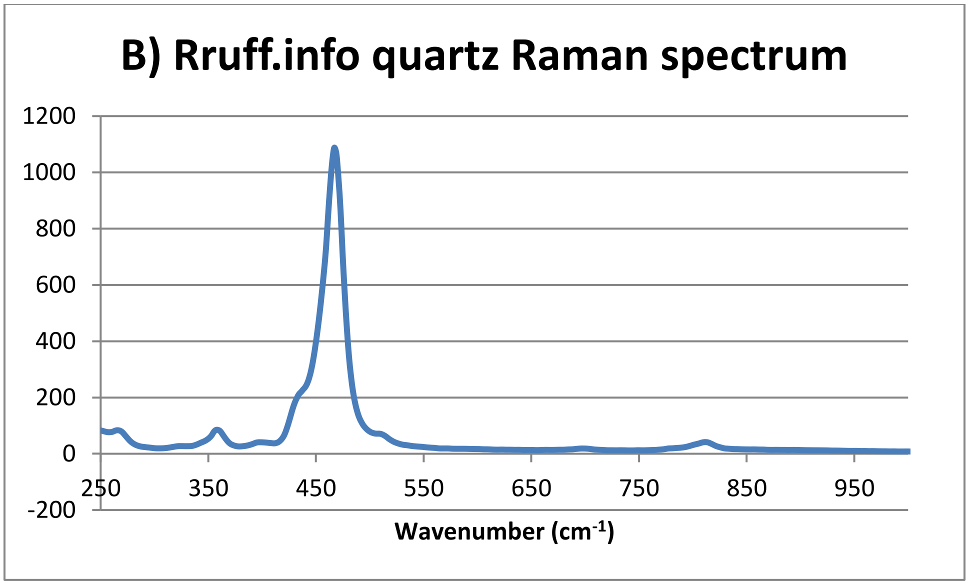

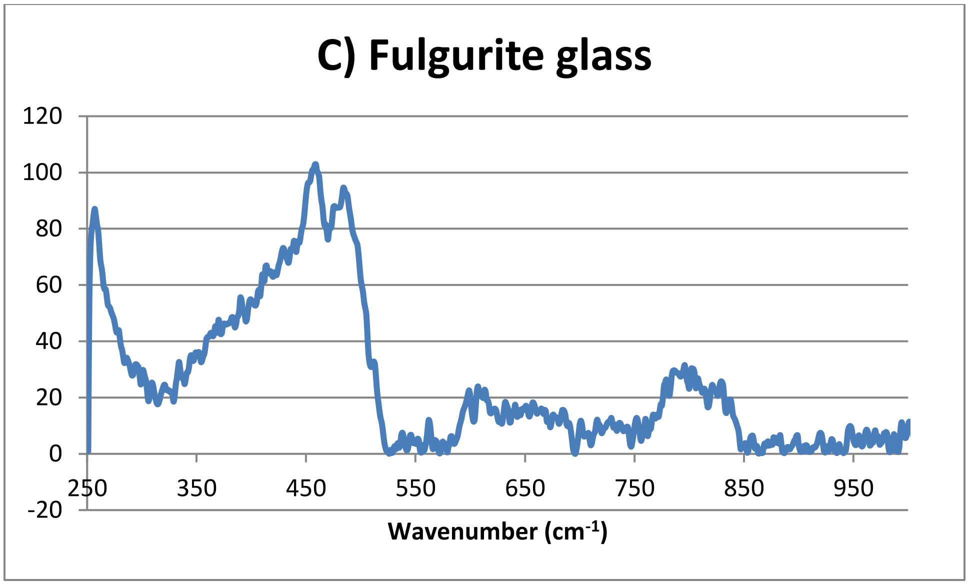

C) Fulgurite glass
120
100
80
60
40
20
0
-20
250
350
450
550
650
750
850
950
Wavenumber (cm-1)

Table S1. ICP-MS analysis of partial major elements of a Polk County, FL fulgurite fragment.

Oxide	Wt. %
FeO	0.15
MgO	0.04
Al_2O_3	0.7
P_2O_5	BDL
CaO	0.4
SiO_2	98.8

References.

1) Lafuente, B., Downs, R. T., Yang, H. & Stone, N. The power of databases: the RRUFF project. In: *Highlights in Mineralogical Crystallography* (eds. Armbruster, T. & Danisi, R.M.) Berlin, Germany, W. De Gruyter, 1-30 (2015).
2) Haynes, C. V., et al. The Murray Springs Clovis site, Pleistocene extinction, and the question of extraterrestrial impact. *Proceedings of the National Academy of Sciences*, *107*(9), 4010-4015 (2010).
3) Carter, E. A., Hargreaves, M. D., Kee, T. P., Pasek, M. A., & Edwards, H. G. A Raman spectroscopic study of a fulgurite. *Philosophical Transactions of the Royal Society of London A: Mathematical, Physical and Engineering Sciences*, *368*(1922), 3087-3097 (2010).
